# Repair of a right coronary artery rupture with perforated right ventricle following spontaneous pseudoaneurysm: a case report

**DOI:** 10.1186/s40792-024-01941-7

**Published:** 2024-06-12

**Authors:** Masato Furui, Hitoshi Matsumura, Yoshio Hayashida, Go Kuwahara, Masayuki Shimizu, Yuichi Morita, Yuta Matsuoka, Chihaya Ito, Masato Hayama, Kayo Wakamatsu, Hideichi Wada

**Affiliations:** 1 Cardiovascular Surgery Department, Fukuoka University Hospital, 7-45-1, Nanakuma, Jonan-ku, Fukuoka-city, Fukuoka 814-0180 Japan; 2 Emergency Department, Fukuoka University Hospital, 7-45-1, Nanakuma, Jonan-ku, Fukuoka-city, Fukuoka 814-0180 Japan

**Keywords:** Cardiac tamponade, Coronary artery bypass grafting, Coronary artery rupture, Fistula, Pericardial adhesion, Post-inflammatory adhesions, Pseudoaneurysm, Ventricular perforation

## Abstract

**Background:**

Following the rupture of a coronary artery, a patient’s condition usually deteriorates rapidly due to cardiac tamponade. A pseudoaneurysm due to a coronary artery rupture is rare; however, when a spontaneous coronary artery pseudoaneurysm occurs without tamponade, it creates a fistula in the right ventricle, often requiring surgical repair.

**Case presentation:**

This report describes the case of a 68-year-old man who presented with chest discomfort after a 12-day course of antibiotic treatment for bacteremia. Following coronary angiography, echocardiography, and enhanced computed tomography, he was diagnosed with a right coronary artery pseudoaneurysm accompanied with perforation of the right ventricle. Severe adhesions were observed during emergency surgery surrounding the entire heart. The patient presented with risk factors for coronary artery disease, including hypertension and smoking history. His coronary artery was severely calcified due to end-stage renal failure requiring dialysis; thus, a covered stent could not fit inside the arterial lumen. Consequently, coronary artery bypass grafting to the right coronary artery and right ventricle repair were performed. Unfortunately, the patient died postoperatively due to sepsis from intestinal translocation. This rare development was hypothesized to be an incidental result of the combination of severe post-inflammatory adhesions, extensive coronary artery calcification, and rupture of the calcification crevices.

**Conclusions:**

In the case of a severe post-inflammatory response, shock without cardiac tamponade may require further scrutiny by assuming the possibility of inward rupture. For patients in poor condition, two-stage surgical treatment might be considered after stabilization with a covered stent.

## Background

A coronary artery rupture is rare; however, death from a rapidly developed, early-onset cardiac tamponade has been previously observed [1]. Pseudoaneurysms most commonly occur secondary to catheter-based interventions, surgical complications, trauma, or infections [2, 3]. Spontaneous coronary artery pseudoaneurysms are even rarer and can be complicated by the pseudoaneurysm penetrating the right ventricle (RV).

Herein, we report an extremely rare occurrence of a coronary artery pseudoaneurysm accompanied by a fistula to the RV caused by a right coronary artery (RCA) rupture. To our knowledge, no such case has yet been reported.

## Case presentation

**Fig. 1:**
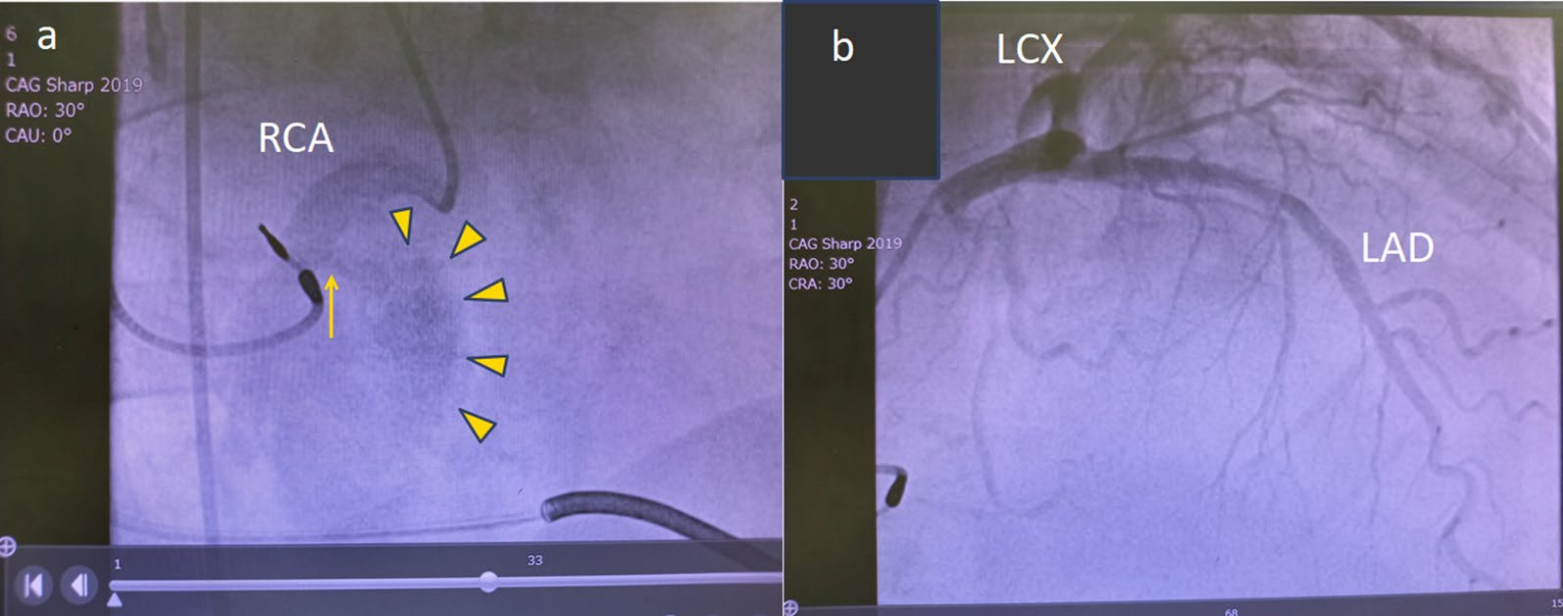
Emergency coronary angiography. **a** Suspected fistula (arrow) originating from the RCA at segment 2 and intracardiac contrast effect (arrowheads) in RAO 30-degree and CAU 0-degree views. **b** No evidence of acute myocardial infarction in the left coronary artery. CAU, caudal; LAD, left ascending artery; LCX, left circumflex artery; RAO, right anterior oblique; RCA; right coronary artery

**Fig. 2:**
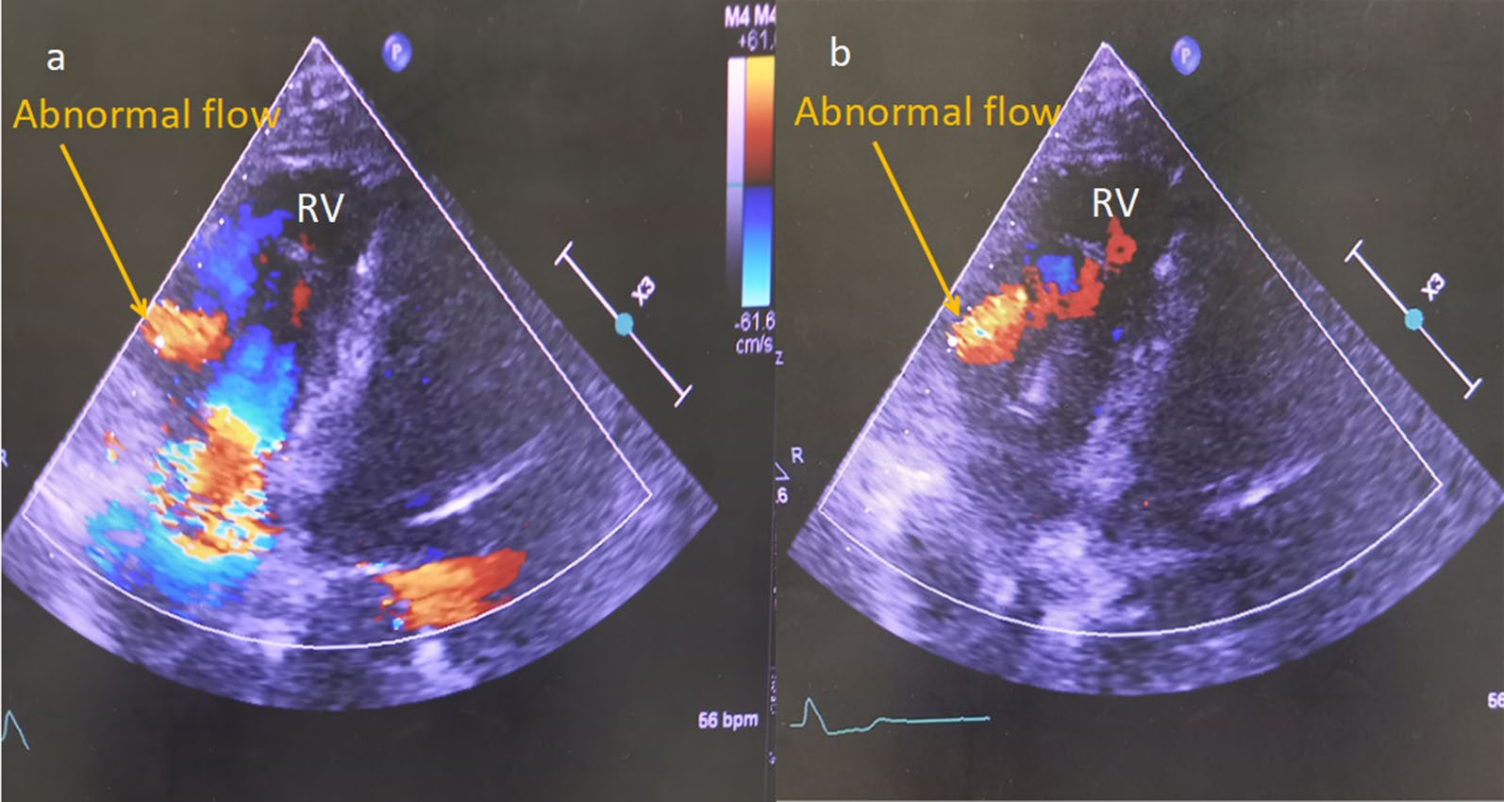
Preoperative transthoracic echocardiography. **a** Abnormal flow (arrow) into the RV observed in the four-chamber view of the systolic phase. **b** Abnormal flow (arrow) into the RV in the diastolic phase. RV, right ventricle

**Fig. 3:**
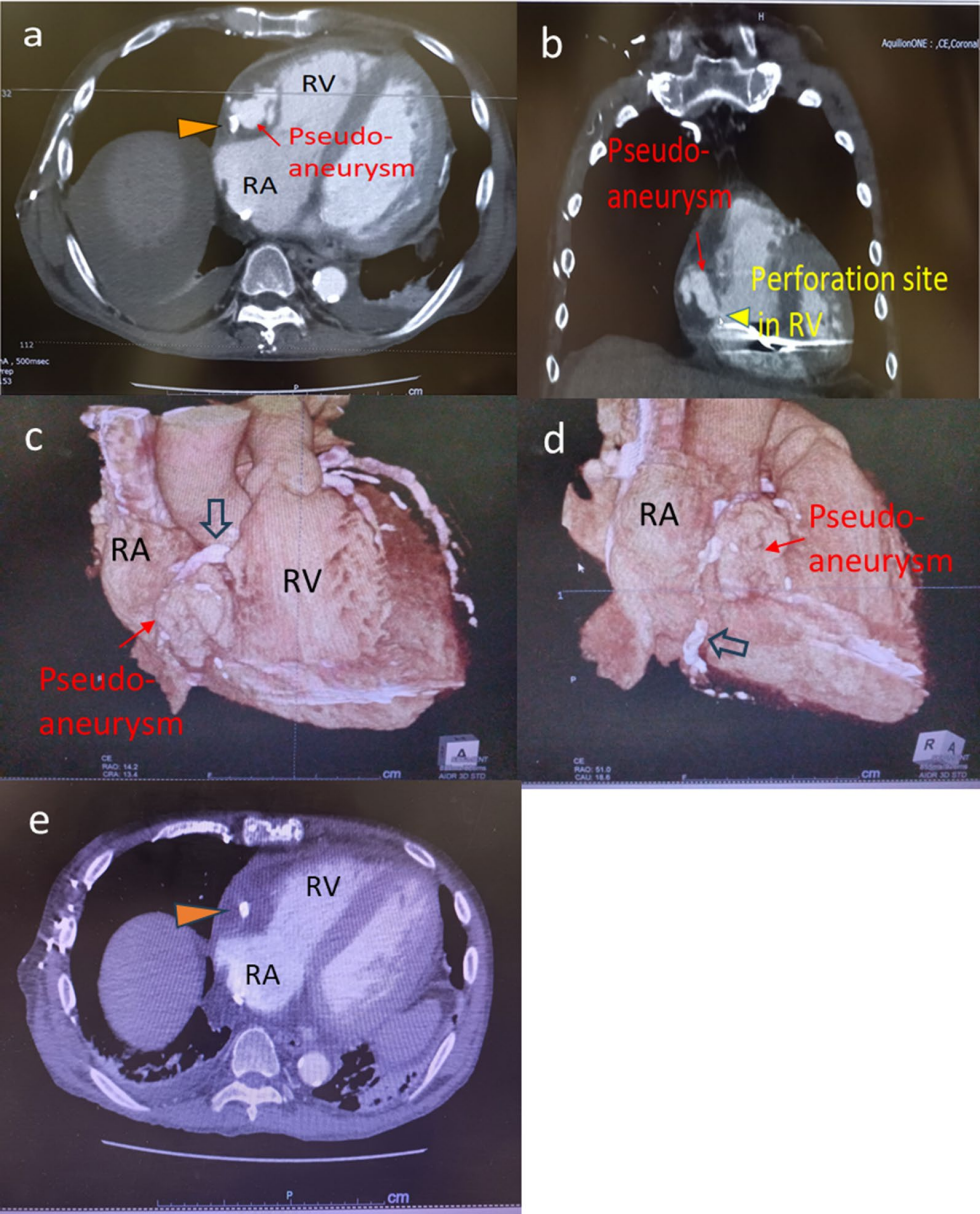
Preoperative enhanced computed tomography. **a** Axial view showing the right coronary artery (arrowhead) with severe calcification around the pseudoaneurysm (arrow). **b** Coronal view showing shunt flow to the RV through the pseudoaneurysm (arrow) and the perforated RV (arrowhead). **c** and **d** 3-dimension CT showing pseudoaneurysm measuring 5.5 × 4.0 cm around the RCA with severe calcification (open arrow). **e** Enhanced CT with an axial level equivalent to **a** on admission. No pseudoaneurysm was seen around the calcified RCA (arrowhead). CT; computed tomography; RA, right atrium; RCA, right coronary artery; RV, right ventricle

A 68-year-old man complained of fever and cervical pain and visited the emergency department. He had several comorbidities and his medical history included polycystic kidney disease, end-stage renal failure treated by regular hemodialysis, chronic heart failure with low ejection fraction, cardioverter defibrillator implantation, paroxysmal atrial fibrillation, hypothyroidism, and chronic inflammatory demyelinating polyneuropathy treated with steroids. Due to many risk factors, he was suspected of having either bacteremia resulting from an infection acquired during vascular access, or meningitis due to cervical pain. He was admitted for secondary sepsis because a blood test indicated elevated inflammatory marker levels: white blood cell count, 11,800/µL; C-reactive protein, 16.6 mg/dL; and procalcitonin, 28.7 ng/mL. No heart murmur was audible, and subsequent computed tomography on admission revealed no suspected sources of infection. Therefore, we administered meropenem and vancomycin empirically. Blood culture on admission led to the identification of methicillin-susceptible *Staphylococcus aureus*. Based on the culture result, we replaced meropenem with cefazolin on day 4. Echocardiography after admission did not yield definitive findings (namely, vegetation) for infective endocarditis. The patient recovered following antibiotic therapy, as indicated by a white blood cell count of 6500/µL and a C-reactive protein level of 1.7 mg/dL; his subsequent blood culture results were negative. However, 12 days after admission, the patient complained of sudden chest discomfort, and subsequently, his systolic blood pressure dropped to 60 mmHg. Emergency coronary angiography (CAG), performed due to the patient’s troponin I level of 2900 ng/mL, indicated the presence of a fistula originating from the RCA and perforating the RV at segment 2 (Fig. 1). Transthoracic echocardiography revealed abnormal flow into the RV (Fig. 2). After the patient’s vital signs were temporarily stabilized via volume addition, enhanced computed tomography (CT) revealed a rupture of the RCA and a fistula in the RV caused by the pseudoaneurysm around the RCA (Fig. 3a–d). The patient became unstable; hence, emergency surgery was performed.

**Fig. 4:**
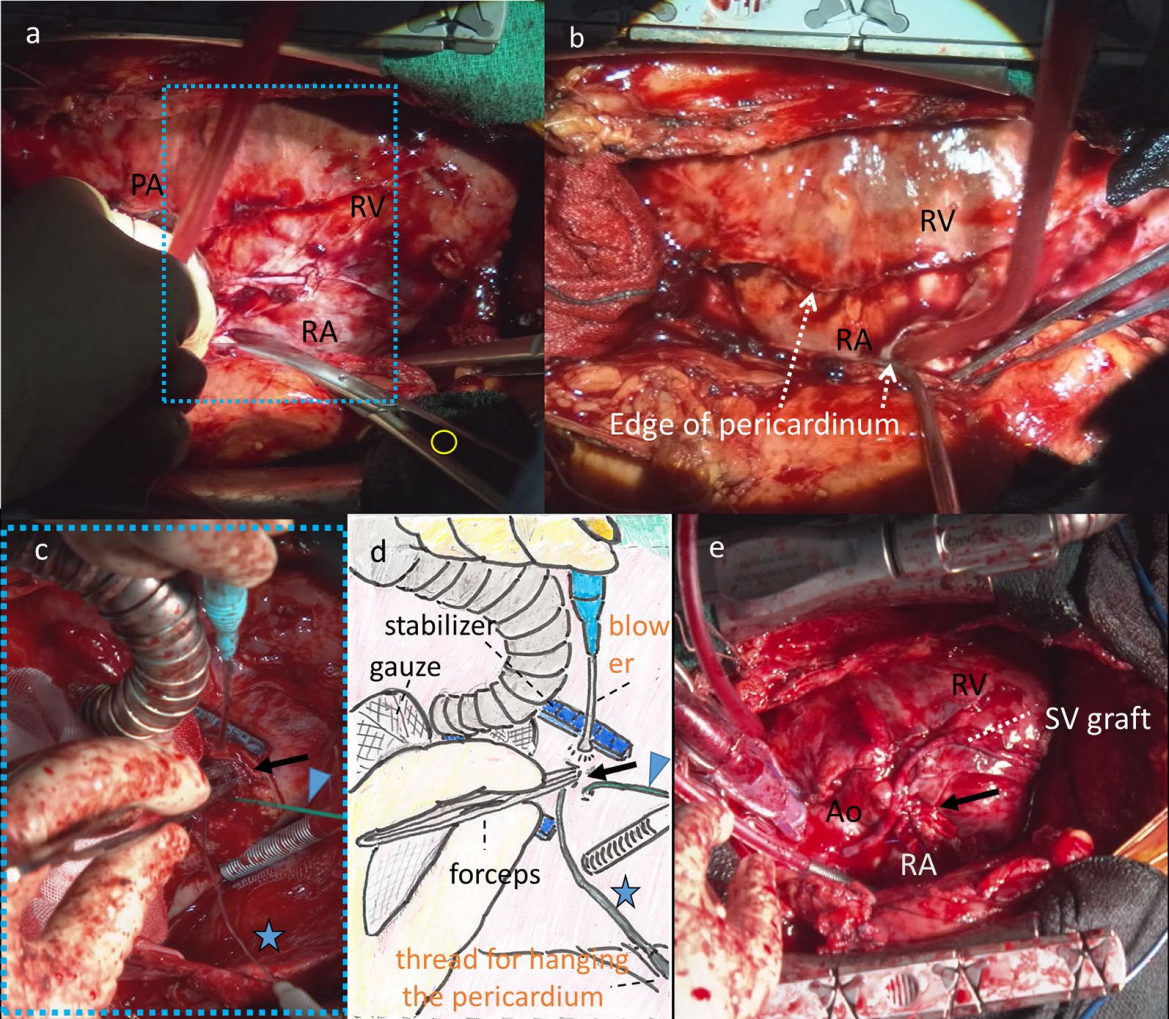
Operative images. **a**, **b** and **e** equivalent views of the operative field. **c** and **d** schema of **c** showing the area corresponding to the blue dotted square in **a** after opening the pseudoaneurysm. **a** Image during pericardial dissection using Metzenbaum scissors (open yellow circle). Note the severe circumferential adhesions and diminished pericardial space. **b** Barely dissected pericardial edges can be seen in the close-up image of **a**. Note the absence of a pericardial cavity due to severe adhesions. **c** and **d** Surgical exploration demonstrating the RV wall with forceps or sonde (star) inserted into the perforation (arrow) after resecting calcified RCA around segment 2. Distal RCA was temporarily blocked by the green occlusion balloon (arrowhead). **e** Completion image after aortocoronary bypass with an SV graft. The perforation site at the border of RA and RV was repaired (arrow) using monofilament with pledget. Ao, aorta; PA, pulmonary artery; RA, right atrium; RCA, right coronary artery; RV, right ventricle; SV, saphenous vein

After median sternotomy, extremely severe adhesions of the entire heart surface and the pericardium were evident, with minimal pericardial effusion (Fig. 4a, b). First, coronary artery bypass to the posterior descending branch at segment 4 was performed via the saphenous vein while the patient was on cardiopulmonary bypass. Next, the ruptured RCA and perforated RV were repaired by ligation of the RCA with endarterectomy due to severe calcification and by suturing with a pledget at the perforation site (Fig. 4c–e). Identification of the RCA by inspection was difficult due to the surrounding inflammatory alterations. Therefore, superficial sonography of the heart and palpation of coronary arterial calcification guided the surgery. Although the patient was weaned from cardiopulmonary bypass, intra-aortic balloon pump insertion was required to treat the persisting cardiogenic shock. The pathological examination of the resected RCA wall, using periodic acid-Schiff, Gram, and Grocott’s stains, revealed no bacteria.

Inotropic support was gradually weaned postoperatively. Enhanced CT on postoperative day 5 did not reveal any complications regarding the cardiac surgery and the patency of the graft; however, paralytic ileus was in development. The patient’s condition worsened, and he developed sepsis from intestinal translocation (complicated by disseminated intravascular coagulation) on postoperative day 6. Therefore, the antibiotic agent was switched from cefazolin to meropenem, vancomycin, and micafungin to treat the sepsis. Unfortunately, the patient died within 17 h of sepsis development, despite the intensive care administered (including continuous hemofiltration, a blood transfusion, and inotropic agents).

## Discussion

Coronary artery aneurysms are rare (with a prevalence of 0.02%), and surgical treatment is sometimes required because of mechanical complications, such as fistulas, compression, or ruptures [4, 5]. Spontaneous ruptures of the coronary artery or coronary artery pseudoaneurysms are rarely observed [2, 4]. The more commonly observed outward coronary artery ruptures can lead to cardiac tamponade. Few cases of coronary artery–ventricular pseudoaneurysm fistulas have been reported [6, 7]. However, to our knowledge, this is the first description of a spontaneous inward rupture due to perforation of the coronary artery pseudoaneurysm into the RV.

If this rupture resulting in pseudoaneurysm was a simple perforation of a healthy lesion, a covered stent would have been the first strategy to implement [2, 8]. However, in this case, considering that the RCA had very severe calcifications (commonly seen in patients with renal dysfunction), the covered stent was a poor fit [9]. An infection around the perforation site was also concerning because of pre-existing bacteremia. Consequently, open surgical repair was considered the reliable and superior strategy in the present case. In hindsight, two-staged surgical repair should have been considered after trying the covered stent, even if it is not sufficient.

The following factors were considered as pathogenic mechanisms: (1) strong adhesions between the pericardium and RCA and diminished pericardial space, resulting from the significant inflammation and adhesions of the surrounding tissues; (2) solid RCA calcification with an uncalcified posterior wall; and (3) fragile RCA posterior and RV walls due to the bacteremia or endocarditis. In the literature, patients with polycystic kidneys are reported to have a higher frequency of coronary artery aneurysms compared to those without this condition [10]. However, the CT scan on admission did not reveal any coronary artery aneurysm, and the subsequent clinical course after admission clearly influenced this outcome (Fig. 3e). Another possible etiology could be an infected aneurysm. However, we might not examine the tissue most suspected of infection, as the right ventricular tissue was not sampled for testing due to concerns about enlarging the fistula. In addition, the prior administration of antibiotics could have masked the infection. Consequently, although the pathological findings suggest the absence of infection, we cannot completely rule out the presence of an infectious aneurysm. Moreover, the possibility of infectious endocarditis cannot be fully dismissed, even though the Duke criteria were not met, given the patient’s status as a compromised host due to hemodialysis and steroid use. In addition, although we conducted transesophageal echocardiography (TEE) once, it would have been prudent to perform TEE several times and conduct culture tests. As a result, we could not definitely determine the cause of the pseudoaneurysm. However, the cardiac tissue was vulnerable, as commonly seen in steroid users. First, as an onset mechanism, the RCA ruptured at segment 2, where calcification was interrupted. Second, what was considered an extravasation appeared as a pseudoaneurysm instead of cardiac tamponade due to severe adhesion to the pericardium. Third, the pseudoaneurysm increased in size and perforated the right ventricle. We presumed that an inward rupture, as presented in this case, would not develop unless some of the predisposing conditions mentioned earlier were met. Echocardiography and CAG assisted in the detection, and enhanced CT was essential for the morphological assessment and understanding, of the pathological condition in this unique case of a cardiovascular shock that developed without cardiac tamponade.

## Conclusion

We observed an extremely rare case of an RCA rupture associated with a perforated RV without pericardial effusion. The unique pathological alterations following significant inflammation may have led to this complication. Several modalities, including echocardiography, CAG, enhanced CT, and superficial sonography, contributed to an understanding of the unique pathological condition and the completion of surgery. For patients in poor condition, two-stage surgical treatment might be considered after stabilization with a covered stent.

## Data Availability

Not applicable.

## Abbreviations

RCA: Right coronary artery
RV: Right ventricle
CAG: Coronary angiography
CT: Computed tomography
